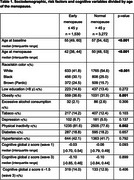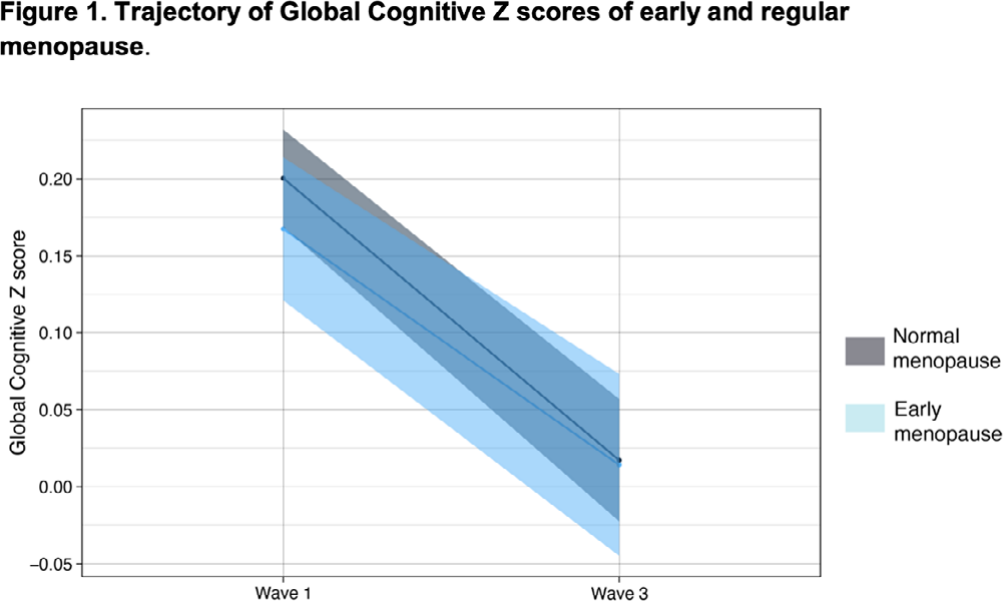# Early menopause and menacme was associated with cognitive decline in ELSA‐Brasil cohort study

**DOI:** 10.1002/alz70860_101998

**Published:** 2025-12-23

**Authors:** Raphael Machado Castilhos, Natan Feter, Ana Luísa Patrão, Sheila Alvin, Bruce Duncan, Maria Inês Schmidt

**Affiliations:** ^1^ Hospital de Clínicas de Porto Alegre, Porto Alegre, Rio Grande do Sul, Brazil; ^2^ Universidade Federal de Pelotas, Pelotas, Brazil; ^3^ Centro de Psicologia da Universidade do Porto, Porto, Porto, Portugal; ^4^ Instituto de Saúde Coletiva da Universidade Federal da Bahia, Salvador, Bahia, Brazil; ^5^ Postgraduate Program in Epidemiology, Universidade Federal do Rio Grande do Sul, Porto Alegre, Brazil; ^6^ Medical School, Universidade Federal do Rio Grande do Sul, Porto Alegre, Brazil

## Abstract

**Background:**

Early menopause (EM) and total time of menstruation (menacme) has recently been studied as potential risk factors for dementia. However, the evidence in the literature shows conflicting results. We aim is to evaluate EM and menacme as risk factors for cognitive decline in the Longitudinal Study of Adult Health (Estudo Longitudinal da Saúde do Adulto, ELSA‐Brasil).

**Method:**

Risk factors, sociodemographic variables and reproductive variables were collected between 2008 and 2010 (wave 1) and the global cognitive function score was established, by averaging Z scores from six standardized tests, approximately 8 years later (wave 3). EM was defined as the age of last menstruation ≤ 45 years at wave 1 and menacme as age at menopause minus age at menarche, in years. The effect of the EM on cognitive decline between waves 1 and 3 was assessed with a mixed linear model (MLM). Women still menstruating at baseline were excluded. All models were adjusted for age and dementia risk factors (low education, hypertension, diabetes, obesity, physical inactivity, depression, alcohol and tobacco consumption).

**Result:**

Of the 15,105 individuals (35‐74 years) of the ELSA‐Brasil, 8218 (54.4%) were female and, of these, 4885 (59.4%) reported having stopped menstruating at wave 1. Of these, 1530 (31.3%) had early menopause. Women with EM were younger (55 [49, 60] vs. 57 [54, 62]), more frequently auto‐identified Black and Brown skin colors and had higher frequencies of obesity and physical inactivity than women without early menopause (Table 1). Total menacme time was 36 [31‐39] years, lower in EM group (29 [25‐31] vs 38 [36‐40]). In the MLM analysis, controlling for confounders, participants with EM had more rapid decline (0.06 z‐scores in 8 years) (Figure 1) and more time of menacme were also associated with more rapid cognitive decline (0.009 z scores in 8 years).

**Conclusion:**

Early menopause and menacme were associated with worsening cognitive trajectories in the ELSA‐Brasil study.